# Efficacy of midwife-led role orientation of birth companions on maternal satisfaction and birth outcomes: a randomized control trial in Uganda

**DOI:** 10.1186/s12884-023-05978-8

**Published:** 2023-09-18

**Authors:** Eva Wodeya Wanyenze, Gorrette K. Nalwadda, Nazarius Mbona Tumwesigye, Josaphat K. Byamugisha

**Affiliations:** 1 Department of Nursing, Mbarara University of Science and Technology, P.O Box 1410, Mbarara, Uganda; 2 Department of Nursing, College of Health Sciences, Makerere University, Kampala, Uganda; 3 Department of Epidemiology and Biostatistics, School of Public Health, Makerere University, Kampala, Uganda; 4 Department of Obstetrics and Gynecology, College of Health Sciences, Makerere University, Kampala, Uganda

**Keywords:** Continuous support, Outcomes, Birth companion, Low-resource setting, Maternal satisfaction, Uganda

## Abstract

**Background:**

The World Health Organization recommends birth companionship for all women in labor. There is insufficient evidence on birth companionship in low-income settings and it is not clear if role orientation impacts effectiveness. The aim of this study was to assess the efficacy of midwife-led role orientation of birth companions of on maternal satisfaction and birth outcomes in a sub-region in Uganda.

**Methods:**

A stepped wedge cluster randomized trial conducted (control *n* = 240), intervention *n* = 235) from 4 clusters. Women who had a birth companion, in spontaneously established labor and, expecting a vaginal delivery were eligible. The intervention was “midwife-provided orientation of birth companions”. The admitting midwife provided an orientation session for the birth companion on supportive labor techniques. The primary outcome was the chance of having a spontaneous vaginal delivery. Assessors were not blinded. Independent t-test and Chi-Square tests were used to assess the differences by study period.

**Results:**

Mean maternal satisfaction rate was significantly higher in the intervention period compared to the control period (*P* > 0.001). High maternal satisfaction levels were noted among the women who were; at the regional referral hospital, younger, first-time mothers, and unmarried (*P* < 0.001). Satisfaction with pain management was rated lowest across study periods. Satisfaction with humaneness was rated highest with a higher score in the intervention period (93%) than the control (79.5%). There were no statistically significant differences in the mode of delivery, need to augment labor, length of labor and Apgar scores.

**Conclusion:**

Midwife-led role orientation of birth companions increased maternal satisfaction. Nevertheless, no significant effect was noted in the mode of delivery, length of labor, Apgar score, and need to augment labor. Findings could inform the integration of birth companions in the admission process of the woman in labor in similar settings.

**Trial registration number:**

NCT04771325.

## Background

There has been progress in the maternal and neonatal mortality rates in Uganda. This has been mainly attributed to skilled birth attendance [1]. Promoting facility births without making efforts to improve quality of care might be inappropriate. A positive childbirth experience is a significant end point for all women undergoing labor [2, 3]. The intrapartum period is a favorable time to provide women with respectful, individualized, and effective clinical and non‐clinical practices to optimize birth outcomes for the woman and her baby [4]. Numerous labor practices have been previously applied to initiate, accelerate, terminate, regulate the physiological process of labor with the aim of improving outcomes for women and babies [5, 6].

The World Health Organization (WHO) recommends that every woman be supported continuously throughout labor by a companion of choice to promote a positive childbirth experience [5, 6]. Continuous labor support is an important aspect of respectful maternity care that emphasizes that the birthing environment should be emotionally and psychologically safe for the woman and her family [7]. Continuous labor support is defined as the presence of a companion at the bedside of an expectant woman, to coach, empathize with, give practical aid to, and inform the expectant mother about birthing [8]. The companion in this context can be any person chosen by the woman to provide her with continuous support [5, 6]. The effectiveness of continuous labor support has been evaluated mostly in high income countries. Findings show that labor support may improve outcomes for women and infants. These include; increased spontaneous vaginal birth, shorter duration of labor, and decreased caesarean birth, instrumental vaginal birth, use of any analgesia, use of regional analgesia, low five‐minute Apgar score and negative feelings about childbirth experiences [9].

Several barriers have been identified in the implementation of the presence of a companion of choice at birth in resource strained hospital settings. Amongst these is the absence of clear communication with the companion about their role [10]. WHO recommends that labor companions have an orientation session on supportive labor companionship techniques to ensure that their presence is beneficial to both the woman and her health care providers [3, 11]. Currently, women in Uganda are allowed to have a companion of choice during labor. These companions however do not receive an orientation on what is expected of them [11, 12].

There is a deficit of evidence on the effect of continuous labor support in low-income settings. Also, it is still unknown if training improves the effectiveness of continuous labor support [9]. Training in this context is described as having an orientation session on supportive labour companionship techniques [3]. There is limited evidence on the effectiveness of interventions to promote respectful maternity care or to reduce mistreatment of women during labour and childbirth [13]. Findings from this study might add to the insufficient empirical evidence in low-income settings. The aim of this study was to assess the efficacy of midwife-led role orientation of birth companions on birth outcomes and maternal satisfaction in Eastern Uganda. We hypothesized that giving an orientation to birth companions increases the chance of having a vaginal delivery, shortens labor, improves Apgar score and improves maternal satisfaction.

## Methods

### Study setting

The study was carried out in the Bugisu sub-region located in the Eastern part of Uganda. The Bugisu sub-region consists of six districts, including Manafwa, Mbale, Bududa, Sironko, Namisindwa, and Bulambuli. According to the Uganda National Bureau of Statistics, the sub-region is home mainly to the Gisu people with an average household size of 4.8 and a literacy rate of 51.5% [14]. The sub-region has several health facilities, including one district hospital (Bududa) and one regional referral hospital in Mbale district. The Health Centre IVs (HCIV), district hospitals, and referral hospitals at the time had a monthly average of 100, 200, and 600 deliveries respectively. The HC IVs have an average of 12 midwives with one to two midwives per 8-h shift while the district and referral hospitals have about 18 midwives with two to three midwives per shift.

### Design

**Fig. 1:**
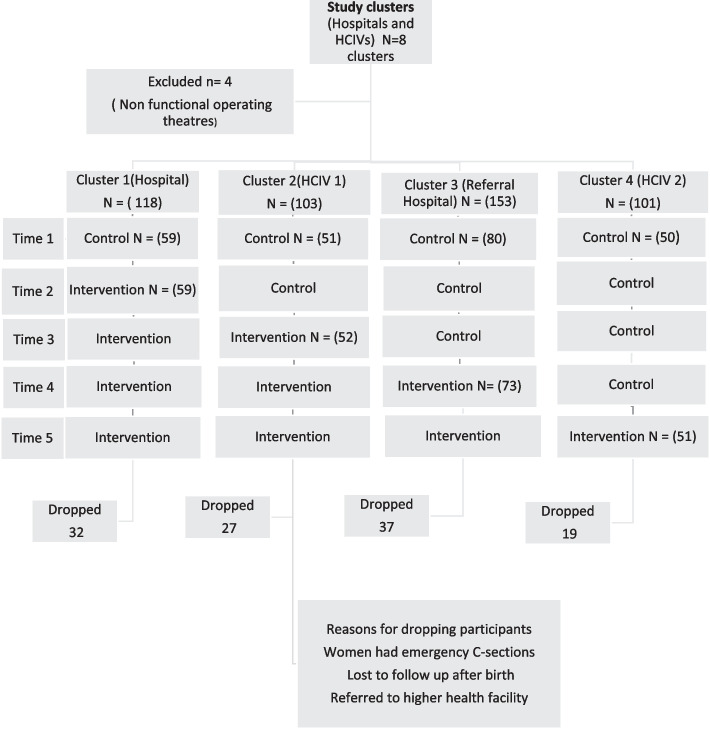
Trial flow chart [16]

A cross-sectional stepped wedge cluster randomized trial was used. In this design, different individuals in the control and intervention are used with a single observation of outcomes [15]. This approach was selected because of the anticipated difficulty in simultaneously introducing the intervention to the different clusters. Additionally, it was preferred for ethical purposes; that is, not to withhold a beneficial intervention from some clusters. For purposes of this study, each selected facility was labeled as a cluster. The intervention was rolled out sequentially to the facilities over 12 months. The facilities were their controls hence buffering the effects of heterogeneity between health facilities. In the first-time block, all clusters were in the control phase and by the last time block, all clusters were in the intervention phase (see Table 1 and Fig. 1). The trial was registered at the U.S. National Library of Medicine ClinicalTrials.gov trials on 25/02/2021 (NCT04771325).

**Table 1:** Trial dates [16]

	**Time 1**	**Time 2**	**Time 3**	**Time 4**	**Time**
Bududa	Control **Jan-Feb 2020**	Intervention **March 2020**	Intervention	Intervention	Intervention
Manafwa	Control **Jan-Feb 2020**	Control **March 2020**	Intervention **Oct-Nov 2020**	Intervention	Intervention
Mbale	Control	Control	Control Oct-Nov 2020	Intervention **Dec-Jan2020**	Intervention
Muyembe	Control	Control	Control Oct -Nov 2020	Control Dec 2020	Intervention **Feb 2021-Mar 2021**

Women who had a birth companion, in spontaneously established labor and expecting a vaginal delivery were included. Exclusion criteria were women with multiple pregnancies, previous cesarean section, with mental illness, deaf or mute [16].

The intervention was “midwife-provided orientation of birth companions”. After labor was confirmed, in addition to the routine admission procedure, the midwife explained to the birth companion the different support techniques and clarified what was expected of them. We assumed that providing an orientation session was likely to boost birth companion confidence, hence increasing the effectiveness of continuous labor support. The content for the orientation session consisted of providing emotional and physical support. Emotional support included being present, demonstrating a caring and positive attitude, saying calming verbal expressions, using humor, and praise, and encouraging and acknowledging efforts. Physical support included supporting her to change positions favoring upright positions, walking with her, giving her drinks and food, massaging, reminding her to go and pass urine, helping her find a comfortable position for pushing, and wiping her face with a cool cloth. The content was developed based on the literature on labor companionship techniques. The orientation sessions were headed by four registered and licensed midwives. These directed and supervised the admitting midwives in the four respective clusters on the orientation of birth companions on labor supportive techniques. The first author EWW trained these midwives on how to conduct these sessions. Orientation was conducted face-to-face, individually for the birth companion. This orientation was integrated into the admission procedure for the woman and lasted about 20 min. Each task was explained in simple terms, including why the task was important and how it was performed, then companions were shown how it was performed, with return demonstrations from the companion. This was repeated for the birth companion to grasp and retain the task [16].

#### Control (usual care)

Women are escorted to the health facilities by one or more family members or friends. One person is allowed besides her to provide support. The support persons do not receive any orientation sessions and have no designated roles. Information is provided on the need arise basis. That is; when the midwife needs anything in particular or help from the birth companion. Routine analgesia is not given. Midwives, medical officers, and obstetricians provide skilled care. Typically, two to three midwives are allocated per 8-h shift managing about six laboring women at a given time.

#### Outcomes

The primary outcome was the chance of having a spontaneous vaginal delivery. The secondary outcomes were; the incidence of having a spontaneous vaginal delivery, length of labor, Apgar score, coping, anxiety and maternal satisfaction. Maternal anxiety and coping during labor are reported in another article and can be accessed at https://doi.org/10.3390/ijerph20021549.

**Fig. 2:**
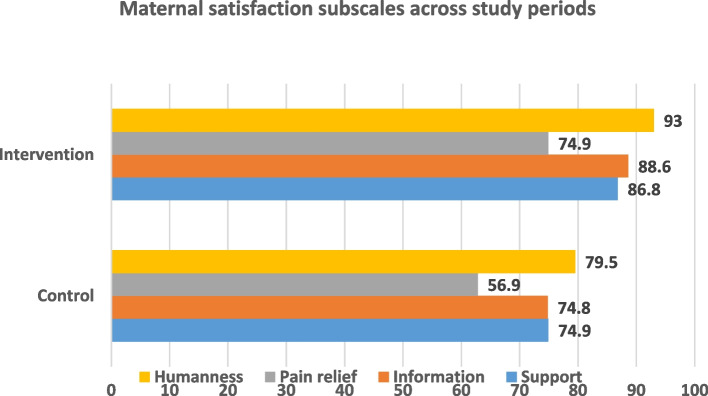
Maternal satisfaction across study periods

Sample size and randomization: The sample size for this trial was calculated based on the primary outcome (incidence of having a spontaneous vaginal delivery). The baseline rate for having a spontaneous delivery was 87%. We assumed that guiding birth companions on continuous labor support contributed to a 10% difference (minimally relevant difference between the two groups as 0.1). We set Alpha at 95% CI (0.05 = 1.96), β multiplier for 80% at 0.842. A sample size of 290 participants per period was calculated. The number of study participants was selected proportionately; that is, according to the patient volumes of the particular facilities. Approximately 12,500 women delivered during the study period including the 6 months break of the COVID-19 lockdown (see Fig. 1). These also included women admitted in second stage, women who gave birth on their way to the hospital, elective cesarean sections, and complicated cases from lower health centers. In total, 580 eligible participants were recruited for the study. See details in Fig. 2.

Randomization for stepped wedge trials is not performed individually but rather involves the crossover of clusters from control to intervention until all clusters are exposed [17]. Using a simple random technique, the principal investigator EWW generated a random sequence of the four hospitals. Numbers 1, 2, 3, and 4 were assigned to the different facilities (Mbale 1, Bududa 2, Muyembe 3, Manafwa 4). Using a random sequence generator, a sequence of “2, 4, 1, and 3” was generated. This sequence is what guided which facility crossed over first to the intervention period. Individual women who met the inclusion criteria were recruited from the clusters by study period. In cluster randomization trials, the intervention targets the cluster to prevent potential contamination of the control arm of study. There was a high chance of birth companions sharing what the midwives had shared with them with the other companions, leading to the need to randomize by cluster. Individual women were recruited because individual level outcomes were assessed [18, 19].

Data were collected from the woman’s labor clinical notes using a data abstraction form. The first part entailed the woman’s socio-demographic information and obstetrical information including; parity, weeks of gestation, status of membranes on admission and birth weight. The second part contained the time of admission and cervical dilation on admission, time of delivery, duration of 1^st^, 2^nd^, and 3^rd^ stage in minutes, whether labor was augmented, Mode of delivery (SVD/CS/Instrumental VD) and Apgar score. Maternal Satisfaction was assessed using a questionnaire; this questionnaire was developed basing on literature on maternal satisfaction with intrapartum care [20–22]. It had four subsections including: socio-demographic and obstetrical information, prenatal history, events of labor and the levels of satisfaction on support, information, pain control, humaneness and general satisfaction with birthing experience. The first author (EWW) pilot tested the tool for reliability and integrated learnings in the tool. Maternal satisfaction was assessed by research assistants after birth on the morning of discharge at the postnatal unit [16].

### Data management and analysis

Data was entered using excel and imported to STATA 14 for analysis [23]. Birth outcomes and maternal satisfaction of women in the intervention period was compared to those in the control period. Participant baseline characteristics were summarized using frequencies and percentages. Statistical tests including Chi-squared tests, t- tests and confidence intervals were two sided. The statistical significance was set at 5%. For each continuous outcome, the mean and standard deviation for each allocated group was presented at 95% confidence interval for the difference and *P* value. For the binary outcomes the percentages and frequencies were presented for each group; including the odds ratio and *p*-values. A *P*-value of < 0.05 was taken to be statistically significant. Subgroup analysis was also done to evaluate treatment effects for specific end point groups [24]. Multivariable analysis was also done to assess the relative contributions of different factors that could affect outcomes.

## Results

### Participant sociodemographic and obstetric characteristics

The majority of the respondents were in the age group of 15–24 years. Most of the respondents were first time mothers (44.7%). A majority were first time mothers (43.6%). All women had at least attended primary school and only 7.8% had a tertiary education. Forty percent of the women in the control period were admitted at 4 cm cervical dilation while the intervention periods had 60% of the women admitted at 4 cm. Most of the women had mothers and spouses for companions; 40.1% and 33.6% respectively. The overall mean of gestation was 38.3 weeks (see Table 2).

**Table 2:** Participant sociodemographic and obstetric characteristics [16]

**Characteristic**	**Total (%)**	**Control % (No.)**	**Intervention % (No.)**
**Age**^**3**^			
15–24	291(61.7)	50.8 (148)	49.2(143)
25–40	181(38.3)	49.2 (89)	50.8(92)
**Education**			
Primary	252(53.1)	52.4 (132)	47.6 (120)
Secondary	186(39.2)	49.5 (92)	50.5 (94)
Tertiary	37(7.8)	43.2 (16)	56.8 (21)
**Marital status**^**10**^			
Unmarried	99(21.3)	29.3 (29)	70.7 (70)
Married	366(78.7)	56.3 (206)	43.7 (160)
**Support person**^**14**^			
Spouse	155(33.6)	60 (93)	40 (62)
Mother	185(40.1)	46.5 (86)	53.5 (99)
Sibling	103(22.3)	36.9 (38)	63.1 (65)
Friend	18(3.9)	55.6 (10)	44.4 (8)
**Parity**			
One	212 (44.7)	51.9 (110)	48.1 (102)
Two	109 (23)	45.9 (50)	54.1 (59)
Three	83(17.5)	50.6 (42)	49.4 (41)
Four or more	70(14.8)	54.3(38)	35.7 (32)
**Gestation weeks**			
Mean (SD)	38.3	38.2 (1.0)	38.3 (1.0)
**Cervical dilatation on admission**			
4 cm	160(33.7)	40 (64)	60 (96)
5 cm	113(23.8)	50.4 (57)	49.6 (56)
6-7 cm	201(42.4)	59.2 (119)	40.8 (82)
**Birthweight**			
Mean	3.2	3.2	3.2
**Cluster**			
Hospital 1	118	50 (59)	50 (59)
HCIV 1	103	49.5 (51)	50.5(52)
Referral Hospital	153	52.3 (80)	47.7 (73)
HCIV 2	101	49.5 (50)	50.5 (51)

^3, 10, 14^Missing data

### Birth outcomes

Spontaneous vaginal delivery rate for all participants was 90.3%. More than half of the women were in labor for 8 h (59%) or less while 32 (8.1%) women were in labor for longer than 16.7 h. The mean maternal satisfaction rate was higher in the intervention period (77.0) compared to the control period (68.1) (*P* > 0.000). There were no statistically significant differences between mode of delivery, length of labor, Apgar score, and the need to augment labor. The length of second stage though not statistically significant was shorter in the intervention period (see Table 3).

**Table 3:** Effect of role orientation of birth companions of on labor outcomes

**Outcome**	**Total**	**Control**	**Intervention**	***p*-value**^**1**^
Mode of delivery				
CS	46 (9.7)	20 (43.5)	26 (56.5)	0.314
SVD	429 (90.3)	220 (51.3)	209 (48.7)	
Total Length of labor (hours)Mean (SD)		8.5 (4.6)	9.2 (4.8)	0.102
1^st^ stage Mean (SD)		8.1 (4.5)	8.8 (4.8)	0.124
2^nd^ stage Mean (SD)		0.5 (0.9)	0.4(0.3)	0.087
3^rd^ stage Mean (SD)		0.2 (0.2)	0.1 (0.1)	0.490
APGAR at 1 min				
> 7	67 (14.2)	32 (13.5)	35 (14.9)	0.652
8–10	406 (85.8)	206 (86.5)	200 (85.1)	
APGAR at 5 min				
> 7	69 (1.9)	6 (2.5)	3 (1.3)	0.322
8–10	464 (98.1)	232 (97.5)	232 (98.7)	
Augmentation				
No	398 (86.9)	205 (87.6)	193 (86.2)	0.647
Yes	60 (13.1)	29 (12.4)	31 (13.8)	
Satisfaction				
Mean (SD)	72.5 (14.7)	68.1 (17.5)	77.0 (9.3)	< 0.001

^*^Denotes statistical significance (*p* < 0.05)

^1^Chi^2^ test

### Subgroup analysis of maternal satisfaction

Sub-group analysis to evaluate treatment effects for specific endpoint groups showed that; the effect was higher among the women who were; at the regional referral hospital, younger, had a secondary level of education, and unmarried (*P* < 0.001). Women who were having their first child, and those who were supported by siblings similarly had a significant increase in maternal satisfaction levels (see Table 4.)

**Table 4:** Effect of role orientation on maternal satisfaction across baseline characteristics

Characteristic	Control Mean (SD)	Intervention Mean (SD)	diff.	*p*-value^1^
**Facility**				
Referral Hospital	65.1(14.6)	81.2(9.7)	16.1	< 0.001
Hospital	75.2(11.6)	81.4(6.8)	6.2	< 0.001
HCIV 1	56(24.7)	67.6(7.7)	11.6	< 0.001
HCIV 2	76.9(7.9)	75.6(3.1)	-1.4	< 0.001
**Age**^a^				
15–24	67.0(18.2)	77.0(8.9)	10.0	< 0.001
25–40	69.3(16.3)	77.2(9.9)	7.9	< 0.001
**Education**				
Primary	68.1(18.4)	76.0(8.4)	7.9	< 0.001
Secondary	67.2(16.5)	78.0(9.0)	10.8	< 0.001
Above secondary	73.1(15.5)	78.3(13.6)	5.3	0.575
**Marital status**				
Unmarried	65.6(18.2)	78.4(8.0)	12.8	< 0.001
Married	68.5(17.6)	76.2(9.7)	7.7	< 0.001
**Support person**				
Spouse	67.4(19.4)	74(8.2)	9.8	< 0.001
Parent	68.7(17.0)	77(8.3)	8.6	< 0.001
Sibling	71.1(14.6)	79.4(11)	8.3	0.046
Friend/relative	64.5(17.5)	79.2(7)	14.7	0.024
**Parity**				
One	66.4(16.9)	77.2(10.0)	10.8	< 0.001
Two	67.2(21.5)	77.5(7.9)	10.3	< 0.001
Three	71.0(17.7)	75.4(9.6)	4.4	0.001
Four or more	71.1(12.8)	77.2(8.6)	6.1	0.025
**Cervical dilatation on admission**				
4 cm	68.7(16.7)	76.5(10.4)	7.8	< 0.001
5 cm	72.8(16.3)	76.5(7.8)	3.8	< 0.001
6-7 cm	65.6(18.2)	77.9(8.9)	12.3	< 0.001

^1^Two sample independent t-test

^a^Missing data

A multivariable analysis was done to assess the relative contributions of different factors that could affect maternal satisfaction. We found a statistically significant difference maternal satisfaction by study period (*p* < 0.000) (see Table 5).

**Table 5:** Multivariate analysis of maternal satisfaction

	**Coef.**	***P*- Value**	**95%CI**
Control period			
Intervention period	7.61	< 0.001*	[4.9- 10.3]
**Age**			
25 and above	0.20	0.917	[-3.4–3.8]
**Education**			
Secondary	1.78	0.212	[-1.0–4.6]
Tertiary	4.08	0.130	[-1.2–9.4]
**Marital status**			
Married	0.07	0.969	[-3.5–3.6]
**Parity**			
Two	1.82	0.309	[-1.7–5.3]
Three	2.15	0.313	[-2.0–6.3]
Four +	3.92	0.129	[-1.2–9.0]
**Cervical dilatation on admission**			
5 cm	2.88	0.112	[-0.7–6.4]
6 cm	-1.19	0.446	[-4.3–1.8]
**Support person**			
Parent	3.96	0.026	[0.5–7.4]
Sibling	5.13	0.007	[1.4–8.8]
Friend/other relatives	1.12	0.755	[-5.9–8.2]

### Maternal satisfaction subscales

Maternal satisfaction was measured across four subscales including; support, information, pain management, and humaneness. The overall satisfaction with support received was higher in the intervention period at 86.8% than the control period at 74.9%. Regarding satisfaction with information received, there was a higher level of satisfaction in the intervention period (88.6%) compared to the control period (74.8%). Satisfaction with pain management was lowest across study periods; women in the intervention periods were more satisfied with their pain management (74.9%) compared to the control period at 56.9%. Overall, satisfaction with humaneness was the highest scored subscale with a higher satisfaction score in the intervention period (93%) compared to the control period at 79.5% (see Fig. 2).

Concerning particular features of care, women were more satisfied with the extent to which birth companions were present, encouraged them to change position and encouraged them in the intervention period (see Table 6). Sixty four percent of the women were satisfied with the massage they received during labor in the intervention period compared to 49% in the control period.

**Table 6:** Aspects of care of maternal satisfaction ratings across study periods

Aspects of care	Control (240)		Intervention (235)	
Ratings	Not satisfied %	Satisfied %	Not satisfied	Satisfied %
**Support**				
The extent to which midwives were present as much as I wanted	10.8	83^6.2a^	11.7	61.7^26.6^
The extent to which midwives encouraged me to move up and change position	27.2	72.8	3.9	96.1
The extent to which midwives helped me relax during contractions	18.7	77.5^3.8^	9.6	90.4
The extent to which my companion was present as much as I wanted	26	74	7.2	90.8
The extent to which my companion encouraged me to move up and change position	31.5	68	9.8	90.2
The extent to which my companion encouraged me	24.1	73.9	12	91.3
**Information**				
I was given information on admission to the maternity unit on where to find specifics	19.9	63.2^16.9^	22.3	77.7
Information on progress of labor	24.2	75.8	6.6	93.4
Information on the health of my baby during labor	19.6	79.6^1.4^	5.3	94.7
The extent to which midwife devoted necessary information and answered your questions	25.1	74.5	8.1	91.9
Health advices on newborn care and breastfeeding	14.6	80.9^4.5^	14.3	85.3
**Pain management**				
The extent to which my pain was assessed	44.8	55.2	27.5	74.9
I was informed about the different methods to relieve my pain	42.4	57.5	41	68
Massage during labor	50.4	49.2	33.6	64.8
I was helped to relax	34.9	63.1	12.5	86.7
Pain relief after birth	40.6	59.4	37.2	80.4
**Humanness**				
Kindness and established trust and understanding by midwives	23.7	80.3	8.5	91.5
Kindness and established trust and understanding by birth companions	20.2	80.3	9.7	91.1
Overall participation of midwives during delivery	15.3	66.8	5.6	94.4
Overall satisfaction with the labor process	16.6	83.4	5.7	95.1

^a^Missing data

Furthermore, 86.7% of the women in the intervention period reported that they were satisfied with the way they were helped to relax during labor compared to the 63.1% in the control period. Concerning humaneness, higher scores were noted regarding kindness, trust and understanding by birth companions in the intervention period (91.1%) compared to (80.3%) in the control period. Surprisingly, we also note that women were more satisfied with the extent to which midwives were present as much as they wanted control group (83%) than the intervention group (61.7%) as shown in Table 6.

## Discussion

In this study, we assessed the efficacy of midwife-led role orientation of birth companions of on maternal satisfaction and labor outcomes. Results from our study showed that the mean maternal satisfaction rate was significantly higher in the intervention period compared to the control period. And, there were no statistically significant differences between mode of delivery, length of labor, Apgar score, and need to augment labor.

The World Health Organization framework for improving quality of care for pregnant women during childbirth highlights experience of care as important as clinical care provision in achieving desired person-centred outcomes [5]. In our study, we found that women in the experimental group were more satisfied with their labor experience compared to those in the control group. Similar findings are reported in a study conducted in Turkey where the women in the experimental group had higher birth satisfaction and had less fear of childbirth [25]. Results from a study done in similar settings showed that the presence of birth companions was significantly associated with satisfaction with basic emergency obstetric services [26]. A related study was conducted to evaluate the effectiveness of an educational manual for companions. Results showed that the women in the intervention group had higher satisfaction with childbirth [27]. Findings from this study revealed that the empowerment of the companion during labor and delivery made a positive difference on the woman’s perception of support [28]. Therefore, we recommend that companions are guided on admission to help them settle into this very significant role to positively impact women’s experience of care.

High maternal satisfaction levels from sub-group analysis were noted among the women who were; at the regional referral hospital, younger, first-time mothers, and unmarried. Better results reported at the referral hospital could be attributed to the seniority of the midwives. Additionally, this was in a fairly urban setting and the effectiveness of the intervention was probably associated with income, and educational level of birth companions [29]. This advantage probably empowered them to comprehend and execute given instructions from the midwives. Regarding first-time and younger mothers, it’s conceivable that they fully trusted birth companions and followed instructions given religiously considering their inexperience**.** Studies show that Prime gravid anticipate labor pain, poor attitudes of health-care personnel and an insecure environment for birth during late pregnancy [30]. Consequently, more practical information in the social support is vital for helping primigravid women approach childbirth positively [31]. In 2016, the institutional delivery rate in the study area was lower (56.2%) compared to national average of 74% [32]. Considering this difference, it is necessary the presence of birth companions be exploited. This could be achieved possibly by providing an orientation session on admission of the woman in labor. We suppose that maternal satisfaction with the experience of care will subsequently influence future reproductive decisions including the place of birth.

Support during childbirth is an important aspect of maternal satisfaction with care. Findings from a study conducted in Uganda showed that women desired the presence of someone most especially when health workers were not available. They needed reassurance, encouragement, and motivation for them to ably navigate through the birthing process [33]. Our study shows that midwives orienting birth companions on basic support techniques increased women’s satisfaction with support received. Another key aspect of maternal satisfaction is pain management. In our study women in the intervention group were more satisfied with the way they were able to relax and the massage that they received compared to the intervention period. Non-pharmacological pain relief methods promote high levels of satisfaction with care. The midwife has a central role in educating the woman and her family to improve experience of care [34]. We also note that women in the control period were more satisfied with the extent to which midwives were present as much as they wanted compared to those in the intervention period. A probable explanation for this is that the midwives perhaps felt the women receiving the intervention did not need them as much and attended to other laboring women. Continuous labor support is a cheap culturally sensitive method of providing labor support and needs to be fostered.

Non-significant findings on birth outcomes are similar to findings from a study where the rates of cesarean section delivery were almost identical in the intervention and control groups. Moreover no significant differences were noted in maternal and neonatal outcomes [35]. The authors hypothesized that the observed result was attributed to the possibility that both groups received the same amount of support. That the usual care group could have received extra support from husbands or partners and family members [35]. Also, a Thai study found no significant differences in the vaginal delivery rate when close female relatives offered emotional and physical support. This finding was ascribed to an insufficient power to find a true difference in their study. Additionally, that those in the experimental group could have exerted less pushing effort since they were supported by the female relatives while the control group also received support from the nurses [36]. Majority of studies however report an increased incidence of a spontaneous vaginal delivery and improved maternal and neonatal outcomes [9].

A possible explanation for the contrast of findings in our study could be attributed to short duration of the intervention. The birth companions in our study had one session of orientation for 20 min on admission; this was probably not enough to make a difference. The current study found that orientation had a greater effect on the psychological outcomes (anxiety, coping and maternal satisfaction) [16]. Probably the emotional support techniques were easier to recall and execute compared to physical support techniques like walking with her, encouraging upright positions and, reminding her to pass urine. Basing on the physiology of labor such support actions have a noteworthy effect on the progress of labor. So, we would recommend more sessions and longer duration of orientation of birth companions. It must also be noted that 60% of the women in our study were admitted at 4 cm in the intervention period while 40% were in the control period. A recent study conducted among 5,606 women in Nigeria and Uganda showed that labor was very slow throughout the early first stage. Labor may not naturally accelerate in some women until a cervical dilatation of 5 cm is reached [37]. This could be a reasonable explanation for the long length of labor in the intervention period.

Whereas the midwife providing an orientation for birth companions had no significant effect on the biological process of labor, it had an immediate positive effect on how women experienced care given. We also recognize that midwives providing a detailed orientation for birth companions may perhaps be a challenge with large numbers of women in labor at a given time. Also, the short time of interaction may limit building good relations with the midwife lessening the effect of the intervention. Midwives could consider group sessions, illustration chats and videos in the admission areas of busy maternity units. Evaluation of the acceptability and perceptions of birth companions and midwives regarding orienting birth companions is essential for implementation. Furthermore, we excluded women with special needs like mental illness, deaf or mute. Future studies could explore birth companionship for such women because they might need even more support during childbirth.

### Strengths and limitations of the study

The selected study design had the following strengths; Firstly, the facilities were their own controls hence buffering the effects of heterogeneity. Secondly, the stepped wedge design limited contamination of the intervention arm considering the socio nature of the intervention. Also, subgroup analysis of treatment effects of clusters and participants provided useful information for specific baseline characteristics of women. These findings could be used to tailor specific interventions for women.

On the other hand, caution should be taken in generalizing the findings. The clusters were few and had a low power to determine true statistical differences. Secondly, observations collected soon after the roll-out of the intervention and observations collected after sometime could have been different which might have affected the overall treatment effect. Furthermore, only one session of orientation was done and this could have lessened the effect of the intervention. Also birth attendant details like sociodemographic characteristics and years of experience were not captured and could have affected the delivery of the intervention.

## Conclusion

In this study, we assessed the efficacy of midwife-led role orientation of birth companions on birth outcomes and maternal satisfaction**.** Our results suggest that role orientation of birth companions increases maternal satisfaction with experience of labor and has no effect on birth outcomes. Findings from this study may be of benefit in informing the implementation of the integration of birth companions in the admission process of the woman in labor in similar low-resource settings. Considering the staggered introduction of the intervention, we recommend that the study be repeated with larger samples and more clusters to assess the overall effect.

## Data Availability

The data that support the findings of this study are available from the first and corresponding author, Eva Wodeya Wanyenze.
